# *Potentilla discolor* Bunge Ameliorates Streptozotocin-Induced Diabetic Nephropathy in Mice by Modulating GAS5 and miR-21 Expression

**DOI:** 10.1155/ije/1394709

**Published:** 2025-05-30

**Authors:** Yan Yang, Wen Qiu, Jiyuan Xiao, Xuejian Hu, Jiang Han, Luxia Jiang

**Affiliations:** ^1^Department of Endocrinology and Metabolism, Lanzhou University Second Hospital, Lanzhou, China; ^2^Department of Pharmacology, Lanzhou University Second Hospital, Lanzhou, China; ^3^Department of Cardiac Surgery ICU, Lanzhou University Second Hospital, Lanzhou, China

**Keywords:** diabetic nephropathy, flavonoid, lncRNA GAS5, miR-21, podocytes, *Potentilla discolor* Bunge

## Abstract

Diabetic nephropathy (DN) is one of the major chronic complications of diabetes. Podocyte injury has been identified as a factor in the progression of albuminuria in DN. *Potentilla discolor* Bunge (PDB), an important Chinese herbal medicine, has strong hyperglycemia- and hyperlipidemia-lowering qualities. However, its effects on DN are unknown. The present study investigated the effects of PDB and flavone, which are the main ingredients in PDB, on DN and the possible mechanism, with a particular focus on the contributions of the lncRNA growth arrest-specific 5 (GAS5) and microRNA-21 (miR-21). Our results showed that administration of PDB (100 mg/kg/d) and flavone (10 mg/kg/d) for 12 weeks significantly alleviated albuminuria and decreased serum creatinine in STZ-induced DN mice. Moreover, renal cell apoptosis was repressed and diabetes-induced pathological alterations were reversed. We also found a significant decrease in GAS5 and PPARα expression, while miR-21 expression was increased in diabetic kidneys and podocytes under high glucose (HG) conditions. Notably, PDB and flavone counteracted these alterations. Mechanistic investigations showed that both GAS5 and PPARα are specific targets of miR-21, and that GAS5 could compete with PPARα for miR-21 binding, alleviating PPARα inhibition. Finally, we confirmed that PDB and flavone treatment remarkably attenuated the altered levels of oxidative stress parameters in HG–exposed podocytes, as well as inflammatory cytokines and profibrogenic mediators in the serum and urine of STZ-induced DN mice, and in the supernate of HG–exposed podocytes. Moreover, the interaction between GAS5 and miR-21/PPARα was required during this process. The results indicated that PDB and flavone exert renal protective effects, at least in part, by regulating the GAS5 and miR-21/PPARα pathways, which is a promising therapeutic target for delaying the onset of DN.

## 1. Introduction

Diabetic nephropathy (DN) is a major microvascular complication of diabetes mellitus (DM), accounting for nearly half of all end-stage renal disease (ESRD) cases [1, 2], and is an important intervention target of ESRD [3]. To date, however, the precise DN pathogenesis is still not fully understood, while no effective drug currently exists to reverse the associated renal damage. Thus, there is an urgent need for the development of novel therapeutic methods to suppress DN progression.

*Potentilla discolor* Bunge (PDB), a perennial herb belonging to the Rosaceae family, is a widely used traditional Chinese herbal medicine (CHM). Emerging evidence suggests that its bioactive components exhibit therapeutic potential in managing DM and its complications, with a favorable safety profile [4–6]. The therapeutic efficacy of PDB is primarily mediated by flavonoids, its major bioactive constituents, which exert a wide spectrum of pharmacological effects. These include amelioration of hyperglycemia and hyperlipidemia, as well as antioxidant, anti-inflammatory, and antiapoptotic activities, as demonstrated in both experimental models and human studies [4, 7, 8]. To date, however, only a handful of studies have described the specific mechanisms underlying these phenomena, with PDB's effects and its corresponding molecular mechanisms in DN unknown. Peroxisome proliferator–activated receptor alpha (PPARα) is a ligand-activated nuclear receptor that functions as a transcription factor, regulating the expression of multiple genes involved in lipid metabolism, insulin signaling, and energy homeostasis [9, 10]. Findings from a recent study showed that PPARα is one of the potential protein targets for 21 antidiabetic components in PDB, suggesting that these active ingredients may play a role in the activation of PPARα to confer hypoglycemic effects [4].

MicroRNAs (miRNAs) have recently emerged as important regulators of DN. Particularly, miRNA-21 (miR-21), which is significantly upregulated in human kidney diseases, plays the most significant role in the management of fibrotic disorders [11–13]. Previous studies have suggested that miR-21 contributes to fibrogenesis and oxidative epithelial damage in the unilateral ureteral obstruction model of renal injury by downregulating PPARα [12], while its silencing reversed PPARα downregulation and subsequently improved CKD-related cardiac dysfunction [14]. Long noncoding RNAs (lncRNAs) are nonprotein-coding transcripts longer than 200 nucleotides that regulate a spectrum of biological functions [15], including renal responses to hyperglycemia and DN progression [16]. Researchers have regarded lncRNA growth arrest-specific 5 (GAS5), originally isolated from mouse NIH3T3 cells [17], as a tumor suppressor in plenty of malignancies. Previous studies have demonstrated that GAS5 is negatively correlated with miR-21 in cancer and osteoarthritis pathogenesis, and this phenomenon is considered a reciprocal repression feedback loop between both factors [18–20].

The present study aimed to explore the nephroprotective effects of PDB and its main component, flavone, in streptozotocin (STZ)-induced DN and high glucose (HG)–induced podocyte injury mouse models, with a focus on whether this effect takes place via regulation of the GAS5 and miR-21/PPARα signaling axis.

## 2. Materials and Methods

### 2.1. Chemicals and Materials

STZ was obtained from Sigma Aldrich, while mouse TGF-β1, interleukin-1β (IL-1β), interferon-γ (INF-γ), tumor necrosis factor-a (TNF-a), and IL-6 ELISA kits were acquired from Neobioscience (Shanghai, China). Luciferase Reporter Assay System kit was purchased from Promega (Madison, WI, USA), whereas TRIzol reagent, SYBR Green Mix, and reverse transcriptase kits were obtained from Invitrogen. Secondary antibodies, goat anti-rabbit and goat anti-mouse, were procured from ZSGB-BIO (Beijing, China). PDB purity (≥ 98%) was determined by high-performance liquid chromatography (HPLC) and then flavones were extracted by the solvent extraction method, with separation using a polyamide column. PDB and flavones were dissolved in 1% carboxymethyl cellulose (CMC) solution prior to animal experiments. For *in vitro* cell experiments, both compounds were completely dissolved in 0.1% dimethyl sulfoxide (DMSO) to prepare a 10 mM stock solution. The stock solution was sterilized and then diluted to various concentrations using the basal medium.

### 2.2. Animals

Male C57BL/6J mice (six weeks of age) were purchased from the Animal Center Affiliated to Lanzhou University, Lanzhou, China. 25 mice were randomly selected and intraperitoneally injected with 35 mg/kg of STZ dissolved in citrate buffer (pH 4.5), while the 6 remaining mice were given a similar dose of citrate buffer to serve as the control (Con). After 5 consecutive days of STZ injection, whole blood was collected from the mouse′s tail vein, following a 12-h fasting period. Mice with fasting blood glucose (FBG) levels higher than 250 mg/dL were considered diabetic and used in subsequent experiments, while those with a value less than 126 mg/dL after injection with normal saline were stratified into the control group. All mice were provided with standard rodent chows and tap water ad libitum. Urine was collected every week, over a 24-h period, for urine volume and urinary albumin analysis. Mice with elevated urinary albumin levels (the urine albumin level in the DN group had a > 10-fold increase compared to the Con group) were regarded as early DN [21]. Next, DN mice were randomly divided into three groups, namely, DN mice administered with normal saline alone (DN group), PDB (100 mg/kg/d, DN + P group), or flavone (10 mg/kg/d, DN + F group). Treatment was via intraperitoneal injection, comprising 100 μL each time every two days for 12 consecutive weeks. Each group comprised 6 mice. FBG levels were measured each week, using an automatic blood glucose monitor, and body weight was also measured. At the end of the treatment period, mice were anaesthetized via pentobarbitone sodium (30 mg/kg; i.p.) injection, blood drawn from cardiac puncture, into Eppendorf tubes and centrifuged at 4000 rpm to obtain serum. Fresh kidney cortices were also immediately harvested and stored at −80°C until further analysis.

### 2.3. Determination of Renal Function

Levels of serum creatinine and urinary creatinine were analyzed by an automatic biochemical analyzer (Beckman Instruments, Inc., Brea, CA, USA) according to the manufacturer's instructions. The urinary albumin was determined using the mouse Albumin ELISA Kit (Elabscience, Wuhan, China). The urinary albumin-to-creatinine ratio (ACR) was calculated as follows: ACR (mg/mmol) = urinary albumin (mg/L)/urinary creatine (mmol/L).

### 2.4. Histopathological Analysis

Kidney samples were fixed in 4% paraformaldehyde, for not more than 24 h, and then preserved in 0.5% paraformaldehyde. They were embedded in paraffin, cut into 5 μm-thick cross-sections, and then stained with hematoxylin and eosin (H&E). Morphological changes in kidney pathology were evaluated under a light microscope (Olympus, Tokyo, Japan).

### 2.5. TUNEL Assay

Kidney tissues were embedded in paraffin, cut into 5 μm-thick sections, and then apoptosis was assayed using the TUNEL detection kit (KeyGen Biotech Co., Ltd., Nanjing, China), according to the instructions. Five nonoverlapping fields were randomly selected in each section, manually counted, and observed under a light microscope to detect apoptotic cells (light brown). Apoptosis rate was calculated as follows: number of TUNEL-positive cells/total number of kidney cells × 100%.

### 2.6. Murine Kidney Podocyte Culture and Treatment

Conditionally immortalized mouse podocyte MPC-5 cell lines were purchased from iCell Bioscience Inc. The cell lines were cultured in Dulbecco's modified Eagle's medium (DMEM) and supplemented with 10% fetal bovine serum (FBS) (Invitrogen, Carlsbad, CA, USA) in a humidified incubator at 37°C containing 5% CO_2_. Subsequent experiments were conducted using differentiated podocytes that had attained at least 70% confluence. MPC5 cells were stratified into the following groups: NC (grown in normal medium with 5.6 mM glucose), HG (grown for 24 h in medium with 25 mM glucose), HG + P (grown for 24 h in medium with 25 mM glucose and 100 μM PDB), HG + F (grown for 24 h in medium with 25 mM glucose and 20 μM flavone), miR-21 mimic (transfected for 6 h with miR-21 mimic, grown for 24 h in medium with 5.6 mM glucose), HG + P + miR-21 mimic (transfected for 6 h with miR-21 mimic, grown for 24 h in medium with 25 mM glucose and 100 μM PDB), HG + F + miR-21 mimic (transfected for 6 h with miR-21 mimic, grown for 24 h in medium with 25 mM glucose and 20 μM flavone), HG + miR-21 inhibitor (transfected for 6 h with miR-21 inhibitor, grown for 24 h in medium with 25 mM glucose), GAS5 small interfering RNA (si-GAS5) (transfected for 6 h with si-GAS5, grown for 24 h in medium with 5.6 mM glucose), HG + P + si-GAS5 (transfected for 6 h with si-GAS5s, grown for 24 h in medium with 25 mM glucose and 100 μM PDB), and HG + F + si-GAS5 (transfected for 6 h with si-GAS5s, grown for 24 h in medium with 25 mM glucose and 20 μM flavone). All experiments included the following untreated controls: control mimic (mimic-NC), control inhibitor (inhibitor-NC), and control siRNA (si-NC) supplied with vehicles (0.1% DMSO).

### 2.7. Cell Transfection

miR-21 mimic, miR-21 inhibitor, si-GAS5, and their corresponding controls were purchased from RiboBio Co., Ltd (Guangzhou, China). miR-21 mimic and miR-21 inhibitor were used to overexpress and silence miR-21, respectively, while the si-GAS5 vector was used to knockdown GAS5 expression. These complexes and their scrambled negative controls (50 nM) with Opti-MEM were transfected into cultured MPC-5 cells using the Lipofectamine 2000 reagent (Invitrogen, USA), according to the manufacturer's protocol. Next, the cells were incubated with fresh 1640 medium, 6 h posttransfection, and then subjected to subsequent analyses. Sequences of the siRNAs, miRNA mimics, and inhibitors used in the present study are listed in Table 1.

### 2.8. Dual-Luciferase Reporter Gene Assay

Wild-type GAS5 (wt-GAS5)/mutant GAS5 (mut-GAS5) or wild-type PPARα (wt-PPARα) 3′-UTR/mutant PPARα (mut-PPARα) 3′-UTR sequences were amplified by PCR and cloned the downstream of the siCheck2 vector (Promega, Madison, WI, USA). These primer sequences are listed in Table 2. Next, human 293T cells were cotransfected with the wt-GAS5/mut-GAS5 or wt- PPARα 3′-UTR/mut-PPARα 3′-UTR reporter gene plasmid together with miR-21 mimic. After 48 h of transfection, activities were measured using a dual-luciferase reporter gene assay kit (Promega, Madison, WI, USA) according to the manufacturer's instructions. Firefly luciferase was used as a reporter gene with Renilla luciferase as a normalized control.

### 2.9. Measurement of Intracellular ROS

Intracellular ROS was detected sensitively by dichlorodihydrofluorescein diacetate (DCFH-DA) (Beyotime, China). In brief, MPC-5 cells were treated with different stimulations, washed, and then further incubated for 45 min with 20 μM of DCFH-DA at 37°C in the dark. Thereafter, the cells were washed with PBS, suspended in 100 μL PBS, and then analyzed via a flow cytometer. The fluorescent signal was quantified at excitation and emission wavelengths of 500 and 530 nm, respectively.

### 2.10. Analysis of Proinflammatory and Fibrotic Markers

Levels of inflammatory cytokines, such as TNF-α, IL-6, IL-1β, and INF-γ, and fibrotic markers, TGF-β1, in serum, urine, and cell supernatant, were measured using commercially available ELISA kits according to the manufacturer's instructions.

### 2.11. Western Blot Analysis

Proteins were extracted from whole cells and kidney tissues using the RIPA lysis buffer (Beyotime, Haimen, China), containing a protease and phosphatase inhibitor cocktail. Protein concentration was determined using the bicinchoninic acid (BCA) protein assay kit (Beyotime, Haimen, China), as per the manufacturer's instructions, an equal concentration of protein lysate (50 μg) in each sample separated on 8%–12% sodium dodecyl sulfate polyacrylamide gel electrophoresis (SDS-PAGE), and then transferred to polyvinylidene difluoride (PVDF) membranes. The membranes were blocked with 5% skim milk and then incubated with anti-PPARα (1:1000, 15540-1-AP, Proteintech), anti-PPARγ (1:1000, 16643-1-AP, Proteintech), and anti-SMAD3 (1:1000, Ab84177, Abcam) primary antibodies at 4°C overnight. The membranes were washed with PBS and incubated with HRP-conjugated secondary antibodies. Signals were visualized with ECL substrates (Millipore, USA), and densitometric analysis was performed using Image J. Protein expression was normalized to that of *β*-actin.

### 2.12. Quantitative Real-Time Quantitative PCR (qRT-PCR)

Total RNA was extracted from kidney tissues or cultured MPC-5 cells using the TRIzol reagent, following the manufacturer's protocol. The RNA was reverse-transcribed to complementary DNA (cDNA) using a high-capacity cDNA reverse transcription kit (4,368,814, Invitrogen). The cDNA was subjected to qRT-PCR using the SYBR Green Supermix by Roche Light Cycler 96 Real-Time PCR system, targeting GAS5, PPARα, and PPARγ genes, with β-actin also included as an internal amplification control. To confirm the expression of miR-21, the primers for miR-21 and U6 were synthesized by RiboBio (Guangzhou, China), and the sequences of miR-21 were not offered. miR-21 expression was detected using All-in-One miR qRT-PCR Detection Kit (GeneCopoeia, China), with U6 included as an endogenous normalization control for miR-21. Gene expression was determined using the 2^-∆∆CT^ method. Gene-specific primers used in the present study were purchased from Shanghai Sangong Corporation and are listed in Table 3.

### 2.13. Statistical Analysis

Statistical analyses of data from all experiments, performed at least 3 times, were performed using GraphPad Prism 8 and the data were presented as means ± standard deviations (SDs). Differences among groups were determined using one-way analysis of variance (ANOVA), followed by Tukey–Kramer post hoc multiple comparisons test. Data followed by *p* < 0.05 were considered statistically significant.

## 3. Results

### 3.1. PDB and Flavone Treatment Improved Functional and Morphological Features of Renal Injury in DN Mice

**Figure 1:**
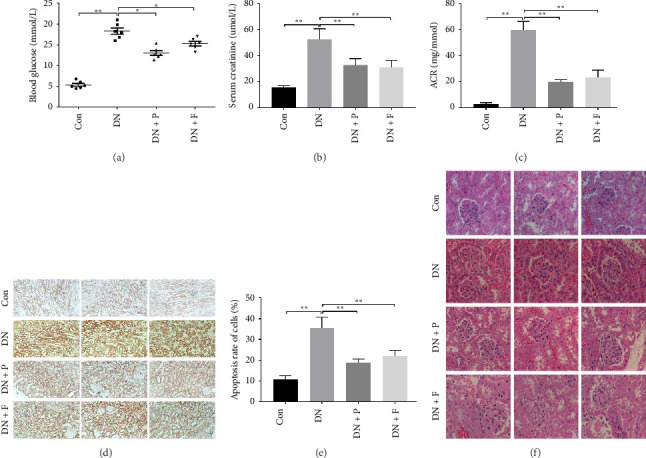
Effect of PDB and flavone on the functional and morphological features of renal injury in STZ-induced DN mice. Data are presented as mean ± SD; *n* = 6 per group. (a) Fasting blood glucose. (b) Serum creatinine. (c) Urinary albumin. (d) Number of apoptotic cells based on the TUNEL assay (magnification: 200×). (e) Summaries of the apoptosis rate among groups in histograms. (f) Morphometric analysis by HE staining in kidney sections (magnification: 400 ×). ^∗^*p* < 0.05 and ^∗∗^*p* < 0.01.

FBG levels of all groups were assessed each week after treatment with or without PDB and flavone. We found that FBG levels were distinctly elevated in the DN group compared to the Con group. However, treatment with PDB and flavone for 12 weeks significantly reduced FBG levels when compared to the DN group (Figure 1(a)). Profiles of effects of PDB and flavone on levels of serum creatinine and urinary albumin in STZ-induced DN mice are shown in Figures 1(b) and 1(c). Summarily, diabetic mice had significantly higher (*p* < 0.01) levels of serum creatinine and urinary albumin than those in the Con group, indicative of DN development. However, treatment with PDB and flavone markedly attenuated (*p* < 0.05) the elevated levels in STZ-induced DN mice, thus preserving renal function.

To assess whether PDB inhibited cell apoptosis in DN, we subjected renal tissues to a TUNEL assay and found a significantly higher number of TUNEL-positive nuclei in the kidneys of mice in the DN relative to those in the control group (35.4 ± 5.25% in DN vs. 10.85 ± 1.58% in the Con group; *p* < 0.01) (Figures 1(d) and 1(e)). In contrast, mice treated with PDB and flavone exhibited significantly lower numbers of TUNEL-positive cells relative to the DN group (22.68 ± 3.76% for the DN + P group, 27.07 ± 5.47% for the DN + F group, vs. 35.4 ± 5.25% for the DN group, *p* < 0.01 and < 0.05, respectively). Collectively, these results indicated that PDB and flavone treatment dramatically alleviated diabetes-induced renal cell apoptosis, while the antiapoptotic effect was more pronounced in the PDB group.

Next, we used H&E staining to determine pathological changes in renal tissues (Figure 1(f)). Results revealed a normal glomerular structure in mice in the control group. Mice in the DN group exhibited histopathological changes in their kidneys, including glomerular hypertrophy, mesangial matrix expansion, tubular epithelial cell vacuolar degeneration, and interstitial inflammatory cells. These were consistent with the characteristics of DN. However, these features were seldom observed in the kidneys of DN mice treated with PDB and flavone. Taken together, these results confirmed that PDB and flavone treatment attenuated renal lesions in STZ-induced DN mice.

### 3.2. PDB and Flavone Downregulated miR-21 Expression in the Kidney of STZ-Induced DN Mice and HG–Induced MPC-5 Cells

**Figure 2:**
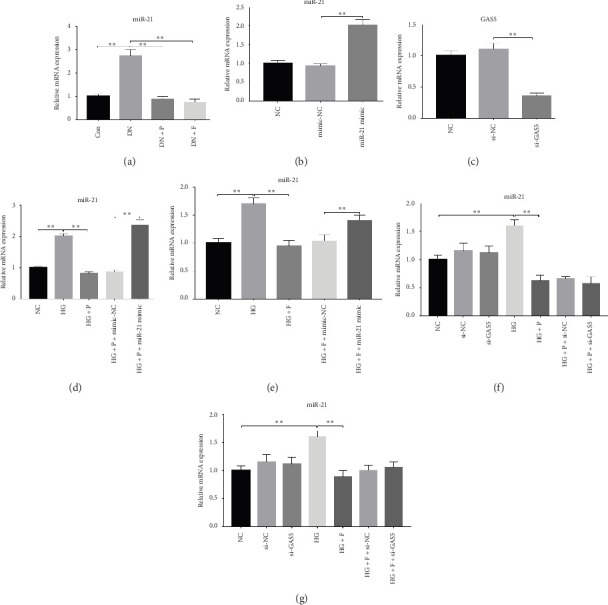
PDB and flavone downregulated miR-21 expression in the kidneys of STZ-induced DN mice and in HG–induced MPC-5 cells. (a) Effect of PDB and flavone on miR-21 expression as observed in the kidney tissue of STZ-induced DN mice using RT-qPCR. (b) Overexpression of miR-21 after transfecting MPC-5 cells with miR-21 mimic as confirmed using RT-qPCR. (c) GAS5 knockdown after transfecting MPC-5 cells with GAS5-siRNA based on RT-qPCR. (d) Effect of PDB on miR-21 expression in HG–induced MPC-5 cells after pretransfection with miR-21 mimic as shown by RT-qPCR. (e) Effect of flavone on miR-21 expression in HG–induced MPC-5 cells after pretransfection with miR-21 mimic, as confirmed using RT-qPCR. (f) Effect of PDB on miR-21 expression in HG–induced MPC-5 cells after pretransfection with GAS5-siRNA, as shown using RT-qPCR. (g) Effect of flavone on miR-21 expression in HG–induced MPC-5 cells after pretransfection with GAS5-siRNA, as shown using RT-qPCR. ^∗^*p* < 0.05 and ^∗∗^*p* < 0.01.

Previous studies have shown that the severity of renal damage is positively correlated with the upregulation of miR-21 expression in biopsy material from DN subjects [22]. Here, we explored the effect of PDB and flavone on miR-21 expression in the kidney of STZ-induced DN mice and found that miR-21 was significantly upregulated (*p* < 0.01) in mice in the DN group relative to those in the Con group (Figure 2(a)). Moreover, PDB and flavone treatment mediated a significant downregulation of miR-21 expression in DN mice (DN + P and DN + F groups) relative to those in the DN group.

Glomerular podocyte injury is a common pathological feature in DN. As expected, the urinary excretion of albumin was elevated, possibly due to podocyte injury in diabetic animals. In contrast, the proteinuria was alleviated by oral supplementation of PDB and flavone to the DN mice (Figure 1(c)). To gain insights into the underlying mechanism of kidney protection conferred by PDB treatment, we determined the relationship between PDB and miR-21 using HG–induced MPC-5 cells as an *in vitro* cell model. First, we transfected MPC-5 cells with miR-21 mimic to successfully overexpress miR-21 (miR-21 mimic group) (Figure 2(b)). Second, we used si-GAS5 to knock down GAS5 expression, before assessing the crosstalk between GAS5 and miR-21 in kidney injury. Transfection efficiency was validated using qRT-PCR (Figure 2(c)). Mice in the HG group exhibited a significant upregulation (*p* < 0.01) of miR-21 compared to those in the NC group (Figures 2(d) and 2(e)). As expected, PDB and flavone treatment significantly downregulated (*p* < 0.01) miR-21 in HG–induced MPC-5 cells (HG + P and HG + F groups) relative to the HG group. Interestingly, the suppression effect of PDB and flavone on miR-21 expression was markedly enhanced after supplementation with miR-21 mimic (HG + P + miR-21 mimic and HG + F + miR-21 mimic groups vs. HG + P + mimic-NC and HG + F + mimic-NC groups, respectively, *p* < 0.01). Notably, we found no statistically significant differences (*p* > 0.05) in miR-21 expression between the si-GAS5 and NC groups. Similarly, there was no significant change in miR-21 expression in MPC-5 cells treated with PDB and flavone (HG + P + si-GAS5 and HG + F + si-GAS5 groups) relative to those in the si-NC transfection group (HG + P + si-NC and HG + F + si-NC groups) (*p* > 0.05) (Figures 2(f) and 2(g)), suggesting that knocking down GAS5 did not affect expression of the miR-21 gene. Collectively, these results indicated that PDB and flavone treatment ameliorate renal injury by downregulating miR-21 expression.

### 3.3. PDB and Flavone Treatment Upregulated GAS5 Expression in Kidneys of STZ-Induced DN Mice and HG–Induced MPC-5 Cells

**Figure 3:**
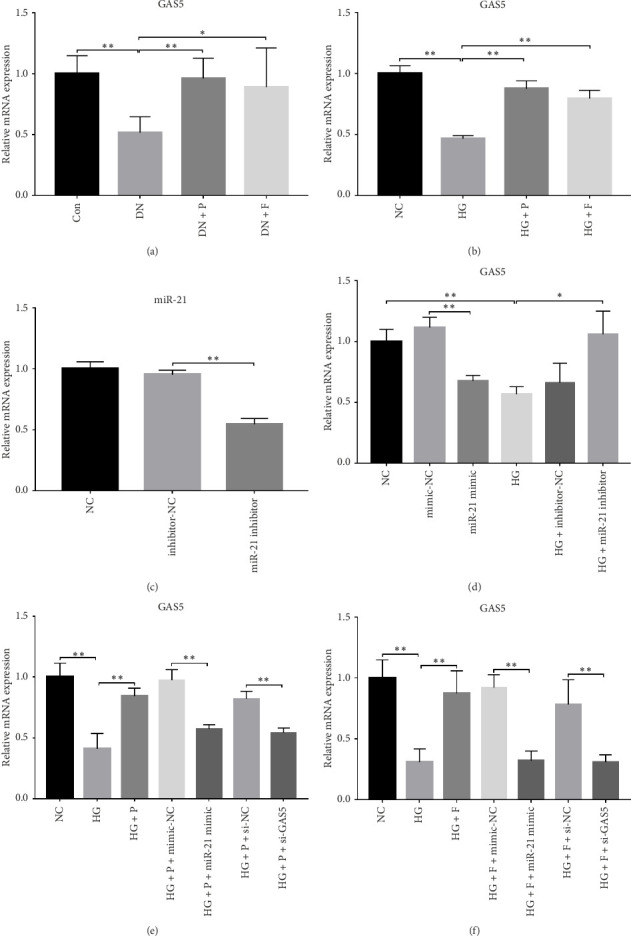
PDB and flavone upregulated GAS5 expression in the kidneys of STZ-induced DN mice and in HG-induced MPC-5 cells. (a) Effect of PDB and flavone on GAS5 expression in the kidney tissue of STZ-induced DN mice, according to RT-qPCR. (b) Effect of PDB and flavone on GAS5 expression in HG-induced MPC-5 cells based on RT-qPCR. (c) miR-21 was inhibited in MPC-5 cells after being transfected with miR-21 inhibitor, which was confirmed using RT-qPCR. (d) After transfected with miR-21 inhibitor or miR-21 mimic, GAS5 expression was observed in MPC-5 cells by RT-qPCR. (e) After pre-transfected with miR-21 mimic or GAS5-siRNA, effect of PDB on GAS5 expression was observed in HG-induced MPC-5 cells by RT-qPCR. (f) After pre-transfected with miR-21 mimic or GAS5-siRNA, effect of flavone on GAS5 expression was observed in HG-induced MPC-5 cells by RT-qPCR.

Results from previous clinical studies have shown that GAS5 might be a key risk factor for DN development [23]. In the present study, we performed qRT-PCR assay to analyze levels of GAS5 expression in kidney tissues and found that this gene was significantly downregulated in mice in the DN, relative to those in the Con group (*p* < 0.01). Conversely, the inhibitory effect of diabetes on GAS5 mRNA expression was markedly reversed by PDB and flavone treatment (DN + P and DN + F groups), as evidenced by 1.9- and 1.7-fold increases, respectively, relative to the DN group (Figure 3(a)). Moreover, GAS5 was significantly downregulated (*p* < 0.01) in HG–induced MPC-5 cells compared to normal cell lines. Intriguingly, PDB and flavone treatment (HG + P and HG + F groups) remarkably upregulated GAS5 expression levels, relative to podocytes treated with HG alone (*p* < 0.01, Figure 3(b)).

A previous study demonstrated that GAS5 expression was negatively correlated with that of miR-21 in breast tumor specimens and cell lines [19]. To explore miR-21's regulatory effect on GAS5, treated with or without PDB and flavone, we transfected MPC-5 cells with miR-21 inhibitor to silence its expression (Figure 3(c)) and then performed qRT-PCR to determine levels of GAS5 expression in response to either miR-21 overexpression or silencing. Results showed that overexpressing miR-21 significantly downregulated GAS5 expression in MPC-5 cells relative to the corresponding negative control group (miR-21 mimic group vs. mimic-NC group, *p* < 0.01) (Figure 3(d)). However, silencing miR-21, GAS5 expression mediated a significant upregulation of GAS5 after HG treatment (HG + miR-21 inhibitor group vs. HG group, *p* < 0.05).

Concomitantly, knocking down GAS5 expression, using si-GAS5 or miR-21 overexpression by miR-21 mimic, abrogated the improved effect of PDB and flavone treatment on GAS5 expression. Moreover, pretransfection of MPC-5 cells with GAS5-siRNAs or miR-21 mimic in combination with PDB and flavone treatment for 24 h significantly downregulated GAS5 expression (*p* < 0.01) under HG conditions (HG + P + si-GAS5, HG + F + si-GAS5, HG + P + miR-21 mimic, HG + F + miR-21 mimic groups, respectively), relative to their corresponding negative control groups (HG + P + si-NC, HG + F + si-NC, HG + P + mimic-NC, HG + F + mimic-NC groups, respectively) (Figures 3(e) and 3(f)).

Overall, these observations indicated that downregulation of GAS5 expression was indispensable in the DN mice and in HG–induced podocytes, implying that this gene plays a crucial role in diabetes microvascular complications. Notably, lncRNA GAS5 is also negatively regulated by miR-21, in a similar fashion, to protein-coding genes. Under such harsh circumstances, PDB and flavone interference upregulated GAS5 expression by downregulating miR-21, suggesting that it has a promising renoprotective effect.

### 3.4. PDB and Flavone Treatment Affected PPARα Expression in Kidneys of STZ-Induced DN Mice and HG–Induced MPC-5 Cells

**Figure 4:**
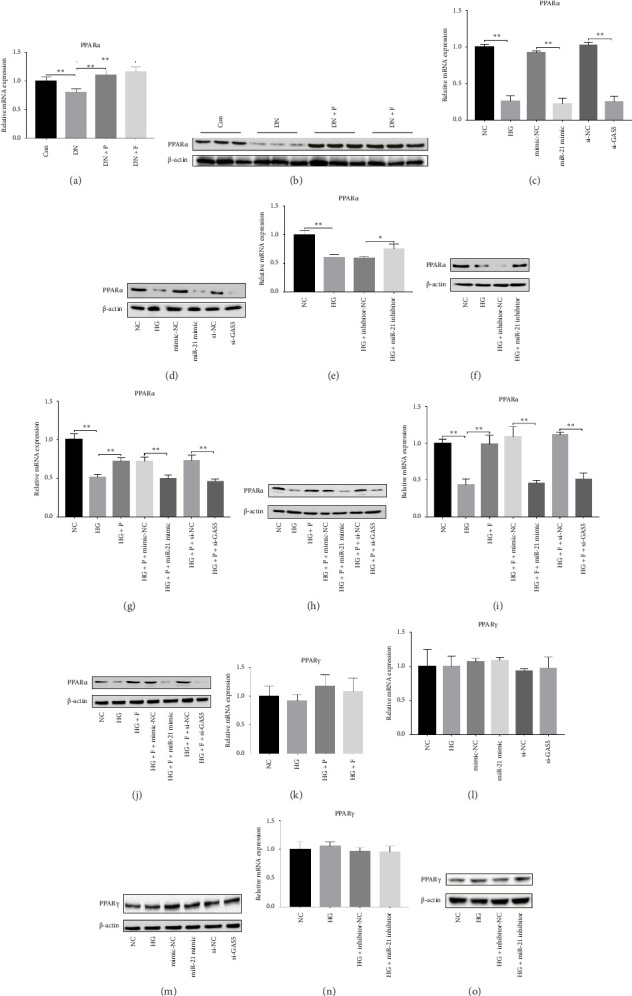
Effect of PDB and flavone treatment on PPARα expression levels in the kidneys of STZ-induced DN mice and HG–induced MPC-5 cells. (a) The mRNA level of PPARα in the kidneys of DN mice as determined using RT-qPCR. (b) The protein expression of PPARα in the kidneys of DN mice as detected using western blot. (c, e, g, and i) The mRNA level of PPARα in MPC-5 cells as determined using RT-qPCR. (d, f, h, and j) The protein expression of PPARα in MPC-5 cells based on western blot. (k, l, and n) The mRNA level of PPARγ in MPC-5 cells based on RT-qPCR. (m and o) The protein expression of PPARγ in MPC-5 cells, according to the western blot analysis. The relative protein levels were calculated using the amount of β-actin protein as reference. ^∗^*p* < 0.05 and ^∗∗^*p* < 0.01.

Results from a previous study suggested that PPARα is not only a target protein for PDB's active components [4] but is also negatively regulated by miR-21 [14, 24]. In the present study, we determined whether PDB regulated the expression of PPARα mRNA and protein in kidneys of DN mice and found that both were significantly downregulated, relative to controls (Figures 4(a) and 4(b)). However, PDB and flavone treatment markedly reversed the expression levels of PPARα mRNA and protein. To further confirm the PDB's underlying regulatory mechanism on PPARα expression, we analyzed levels of PPARα mRNA and protein in MPC-5 cells according to group arrangement. Results showed that both PPARα mRNA and protein were significantly downregulated following either miR-21 overexpression or GAS5 knockdown but upregulated after miR-21 inhibition (Figures 4(c), 4(d), 4(e), and 4(f)). Notably, PPARα expression was significantly downregulated in mice in the HG compared to those in the NC group, although these changes were partially restored by PDB and flavone treatment. However, exposing the cells to miR-21 mimic or si-GAS5 in combination with PDB and flavone attenuated the restoration effect of PDB and flavone on PPARα expression. So, PPARα expression was significantly downregulated in mice treated with PDB and flavone in combination with miR-21 mimic or si-GAS5 relative to their corresponding negative control group under HG conditions (Figures 4(g), 4(h), 4(i), and 4(j)).

Previous studies have shown that PPARγ is also an important member of nuclear receptor superfamily and plays a vital role in exacerbating DN progression [25]. Furthermore, PPARγ and PPARα are reportedly similar with regard to structure and function. In the present study, we analyzed patterns of PPARγ expression, based on group arrangement, and found no significant differences in mRNA and (or) protein levels (*p* > 0.05) between HG–induced MPC5 cells relative to the control group (Figures 4(k), 4(l), 4(m), 4(n), and 4(o)). Similarly, there were no statistically significant differences (*p* > 0.05) in PPARγ expression in response to miR-21 overexpression, GAS5 knockdown, or miR-21 inhibition, suggesting that PPARγ expression has no effect on either miR-21 or GAS5 expression in kidneys of DN mice. Taken together, these results showed that GAS5 and miR-21 have a regulatory effect on PPARα expression even after treatment with PDB, which promotes PPARα expression.

### 3.5. GAS5 Competed With PPARα for miR-21 Binding

**Figure 5:**
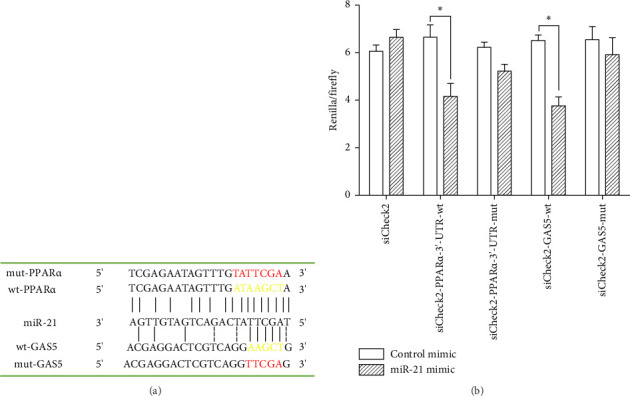
GAS5 and PPARα competed for miR-21 binding. (a) A wt-GAS5 luciferase reporter vector (wt-GAS5), a mut-GAS5 luciferase reporter vector (mut-GAS5) with mutations on the miR-21 binding site in GAS5, a wt-PPARα 3′-UTR luciferase reporter vector (wt-PPARα), and a mut-PPARα 3′-UTR luciferase reporter vector (mut-PPARα) with mutations on the miR-21 binding site in the 3′-UTR of PPARα were constructed. (b) Luciferase activities of PPARα and GAS5 were measured and normalized according to Renilla luciferase activity. ^∗^*p* < 0.05.

We conducted bioinformatics analysis, using online tools (https://www.targetscan.org and https://www.miRCode.org), to align miR-21 and human GAS5 sequences and identify complementary regions constituting a putative miR-21 binding site in the GAS5 gene. Similarly, alignment of the miR-21 sequence with the 3′-UTR of human PPARα revealed a putative miR-21 binding site in the PPARα gene (Figure 5(a)). To further investigate whether both GAS5 and PPARα serve as direct targets for miR-21, and determine whether GAS5 regulates PPARα expression by binding onto miR-21, we constructed a wt-GAS5 cDNA fragment, a mut-GAS5 cDNA fragment with mutations on predicted miR-21 binding site in GAS5, a wt-PPARα 3′-UTR, and a mut-PPARα 3′-UTR with mutations on the miR-21 binding site in the 3′-UTR of PPARα and linked them to reporter vector siCheck2. Next, we cotransfected the luciferase reporter gene vector and mimic-NC/miR-21 mimic into human 293T cells and then performed dual-luciferase reporter gene assay (Figure 5(b)). Results revealed a marked suppression of luciferase activity in both the wt-GAS5 and wt-PPARα luciferase reporter vectors (siCheck2-wt-GAS5 and siCheck2-wt-PPARα) in response to miR-21 mimic transfection relative to that of mimic-NC (*p* < 0.05). Notably, mutations within the binding site of GAS5 (mut-GAS5) or PPARα 3′-UTR (mut-PPARα) abrogated the repression in luciferase activity induced by miR-21 mimic. Finally, we found no statistically significant differences between miR-21 mimic and mimic-NC constructs when either mut-GAS5 (siCheck2-mut-GAS5) or mut-PPARα (siCheck2-mut-PPARα) was used for cotransfection. Collectively, these results showed that both GAS5 and PPARα are specific targets of formiR-21. Notably, GAS5 competes with PPARα for miR-21 binding sites and inhibits endogenous miR-21 function, thereby abolishing repression of PPARα protein expression.

### 3.6. PDB Suppressed HG–Induced ROS Production in Podocytes

**Figure 6:**
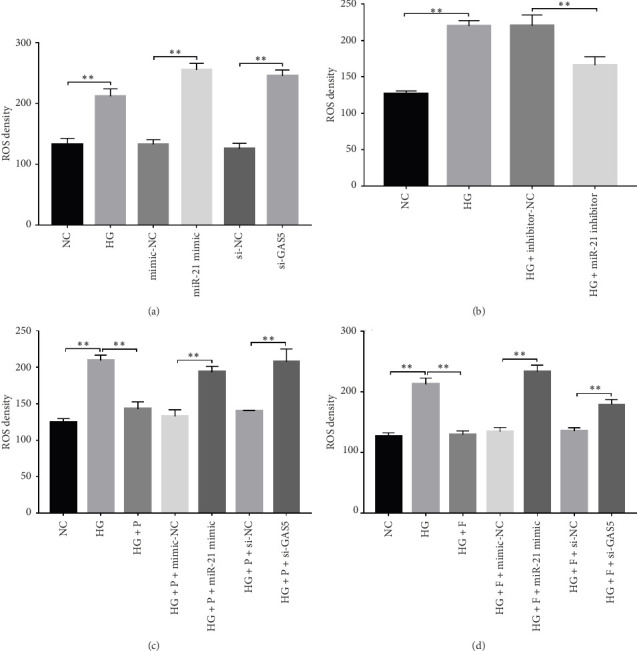
PDB suppressed HG–induced ROS generation in MPC-5 cells. (a–d) The ROS density in podocytes among different groups based on the DCFH-DA assay. ^∗∗^*p* < 0.01.

Generally, ROS plays a key role in DN development and progression. In the present study, we measured intracellular ROS production in MPC-5 cells using the DCFH-DA assay and found significantly higher levels in cells exposed to HG relative to those in the normal glucose group. Conversely, supplement with PDB and flavone significantly inhibited the ROS production in MPC-5 cells (*p* < 0.01, Figures 6(a), 6(b), 6(c), and 6(d)). Moreover, ROS production was significantly suppressed following miR-21 inhibition (HG + miR-21 inhibitor group) but significantly elevated after either miR-21 overexpression (miR-21 mimic group) or GAS5 knockdown (si-GAS5 group), compared to their corresponding negative control groups (*p* < 0.01). However, treatment of cells with PDB and flavone in combination with miR-21 mimic or si-GAS5 pretransfection, markedly attenuated the inhibitory effect of PDB and flavone on ROS production. Therefore, ROS production was significantly higher (*p* < 0.01) in HG + P + miR-21 mimic and HG + F + miR-21 mimic or HG + P + si-GAS5 and HG + F + si-GAS5 groups, relative to their corresponding negative controls (HG + P + mimic-NC and HG + F + mimic-NC or HG + P + si-NC and HG + F + si-NC, respectively). Taken together, these results confirmed that PDB and flavone exert antioxidant effects in HG–induced podocytes, and this effect is partly through modulation of GAS5 and miR-21 expression.

### 3.7. PDB Treatment Downregulated Levels of Fibrotic Markers in STZ-Induced DN Mice and HG–Induced MPC-5 Cells

**Figure 7:**
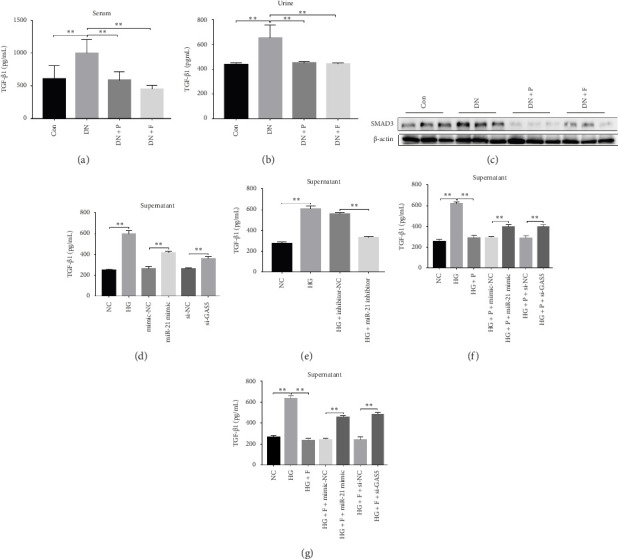
PDB treatment decreased the levels of fibrotic markers in STZ-induced DN mice and HG–induced MPC-5 cells. TGF-β1 concentrations in serum (a) and urine (b) based on ELISA analysis after 12 weeks of PDB and flavone administration in DN mice. (c) The protein expression of SMAD3 in the kidneys of DN mice as detected using western blot. (d–g) The concentrations of TGF-β1 in the supernatant of MPC-5 cells as measured using ELISA. ^∗^*p* < 0.05 and ^∗∗^*p* < 0.01.

To evaluate the effect of PDB on kidney fibrosis in STZ-induced DN mice, we quantified concentrations of TGF-β1 in serum and urine and analyzed the expression of SMAD3 protein in the kidney (markers of fibrosis) after a 12-week PDB and flavone treatment period. Results showed that serum and urine from DN mice had significantly higher levels of TGF-β1 than those of nondiabetic mice (*p* < 0.01). In contrast, PDB and flavone treatment significantly suppressed the increase in TGF-β1 concentrations in DN mice (*p* < 0.01, Figures 7(a) and 7(b)). Similarly, SMAD3 protein was significantly upregulated in mice in the DN, compared to those in the normal control group, whereas PDB and flavone treatment markedly downregulated the expression of SMAD3 protein (Figure 7(c)).

Next, we validated the effect of PDB on kidney fibrosis and then investigated whether GAS5 and miR-21 were involved in the regulatory process. To this end, we collected supernatant of MPC-5 cells and measured TGF-β1 concentrations via ELISA. Results showed that exposure to HG resulted in higher TGF-β1 in MPC-5 cells, although this increase was suppressed following treatment with both PDB at 100 μM and flavone at 20 μM (Figures 7(d), 7(e), 7(f), and 7(g)). These results corroborated the findings from our animal experiments above. Moreover, miR-21 inhibition (HG + miR-21 inhibitor group) mediated a marked reduction in TGF-β1 concentrations, while an opposite effect was observed after miR-21 overexpression (miR-21 mimic group) or GAS5 knockdown (si-GAS5 group), relative to their corresponding negative controls (*p* < 0.01). However, exposure of the cells to PDB and flavone in combination with miR-21 mimic or si-GAS5 markedly attenuated the inhibitory effect of PDB and flavone on TGF-β1 concentrations. Specifically, TGF-β1 concentrations significantly increased (*p* < 0.01) in HG + P + miR-21 mimic and HG + F + miR-21 mimic or HG + P + si-GAS5 and HG + F + si-GAS5 groups, relative to corresponding negative controls. Taken together, these results suggested that the renoprotective effect exerted by PDB and flavone treatment may be partly due to their antifibrotic effect conferred through modulation of GAS5 and miR-21 expression.

### 3.8. PDB Attenuated Renal Inflammation Induced by Diabetes and HG

**Figure 8:**
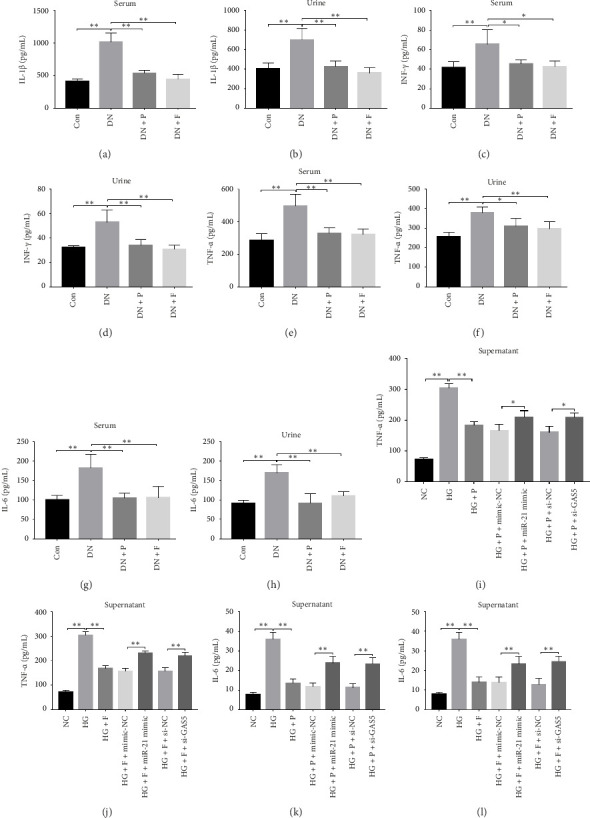
PDB attenuated renal inflammation induced by diabetes and HG. (a–h) The concentrations of serum IL-1β (a), IFN-γ (c), TNF-a (e), and IL-6 (g), and urine IL-1β (b), IFN-γ (d), TNF-a (f), and IL-6 (h) in DN mice after 12 weeks of PDB and flavone administration based on ELISA test. (i–l) The concentrations of TNF-a (i and j) and IL-6 (k and l) in the supernatant of MPC-5 cells as measured using ELISA. ^∗^*p* < 0.05 and ^∗∗^*p* < 0.01.

Chronic inflammation plays an important role in the development and progression of DN. Profiles of the effect of PDB and flavone on levels of inflammatory mediators in serum and urine derived from STZ-induced DN mice are shown in Figures 8(a), 8(b), 8(c), 8(d), 8(e), 8(f), 8(g), and 8(h). Summarily, we found significantly higher levels of IL-1β, IFN-γ, TNF-a, and IL-6 in the serum and urine of mice in the DN, relative to those in the Con group. Interestingly, PDB and flavone treatment significantly inhibited diabetes-mediated increase in levels of IL-1β, IFN-γ, TNF-a, and IL-6. Next, we used ELISA to determine the roles of GAS5 and miR-21 in HG–induced inflammation and found significantly higher TNF-a and IL-6 levels in MPC-5 cells exposed to HG relative to those in the control group (Figures 8(i) and 8(l)). Nevertheless, PDB and flavone treatment remarkably suppressed TNF-a and IL-6 levels, compared to untreated cells. Moreover, it was evident that PDB and flavone treatment had no inhibitory effect on TNF-a and IL-6 levels in MPC-5 cells pretransfected with miR-21 mimic or si-GAS5. Collectively, these results indicated that PDB and flavone conferred kidney protection on STZ-induced DN mice and HG–induced MPC-5 cells by counteracting the elevation in levels of proinflammatory cytokines, through the regulation of GAS5 and miR-21 expression.

## 4. Discussion

In the present study, PDB from *Herba Potentilla SP* prevented renal injury in STZ-induced DN mice and HG–induced podocytes. Moreover, the findings demonstrated that PDB and its main component, flavone, played a protective role in DN by ameliorating renal dysfunction, attenuating renal pathological changes, and suppressing renal oxidative stress, fibrogenic, and inflammatory responses, possibly by modulation of the GAS5 and miR-21/PPARα signaling pathways.

The presence of microalbuminuria due to glomerular dysfunction is a gold standard marker for diagnosing DN in its early stages [26, 27]. Podocytes are highly specialized cells that are critical for maintaining the functions of the glomerular filtration barrier [28]. Studies have suggested that podocyte injury is involved in the pathogenesis of albuminuria in diabetes, and it may be a typical characteristic of early-phase DN [28, 29]. Current therapeutic strategies for diabetic complications primarily target metabolic parameter optimization, including glycemic control, lipid management, and blood pressure regulation. However, the nonspecific nature of conventional therapies can induce undesirable side effects, and the incidence of ESRD due to DN remains high [30, 31]. Chinese herbal medicines containing flavonoid compounds demonstrate renal protective effects in diabetes through multitarget mechanisms, exhibiting favorable safety profiles compared to conventional therapies [32, 33].

Evidence shows that PDB has antidiabetic, hypolipidemic, and antioxidant properties. Thus, we examined the effect of PDB and its main component flavone on renal damage at an early stage using a STZ-induced DN mouse model in the present study. The level of FBG was found to be higher in the DN group than in the control group. A notable decrease was observed in the FBG level of DN mice due to the PDB and flavone treatment. The obtained results are consistent with previous reports in the literature [4, 5, 34].

We found that 100 mg/kg/day of PDB and 10 mg/kg/day of flavone for 12 weeks significantly reduced urinary excretion of microalbumin and serum creatinine levels. Therefore, concurrent treatment of diabetic mice with PDB and flavone, as indicated in the DN + P and DN + F groups, reduced proteinuria production while preserving renal function. Furthermore, the number of TUNEL-positive cells in DN mice was considerably higher than in age-matched nondiabetic controls. However, as compared to the DN group, PDB and flavone treatment remarkably reduced the number of TUNEL-positive cells, inhibited diabetes-induced renal cell apoptosis, and manifested antiapoptotic potential. Apoptosis-induced podocyte loss is a well-known pivotal trigger of progressive glomerular sclerosis [35]. To further verify the kidney-protective effect of PDB, we examined the pathophysiological changes of kidney tissues using H&E staining. The results showed that PDB and flavone treatment reduced glomerular hypertrophy and mesangial matrix expansion and attenuated the histopathological changes consistent with the characteristics of DN. Evidently, these data provide insights into the efficacy of PDB and flavone against renal lesions and podocyte injury.

Subsequently, the mechanism of PDB renoprotection was explored in STZ-induced DN mice and HG–induced mouse podocytes. Reports indicate that GAS5 is associated with the prevalence of DM [36]. Furthermore, GAS5 expression is significantly downregulated in the serum of DM patients and can be used as an efficient biomarker for the diagnosis of DM [23, 36]. In addition, recent studies have identified miR-21 as a potential diagnostic biomarker for the prediction of DN because renal miR-21 is clearly upregulated in DN mice or patients, and pharmacological silencing of miR-21 ameliorates the progression of DN [13, 37]. Therefore, we tested whether PDB treatment could alter the expression of both noncoding RNAs. Under hyperglycemic conditions, both *in vitro* (HG–induced MPC5 cells) and *in vivo* (STZ-induced DN mice), the expression level of GAS5 was reduced, whereas the expression level of miR-21 was elevated. Under the same conditions, PDB and flavone treatment boosted GAS5 expression while inhibiting miR-21 expression, suggesting that GAS5 and miR-21 may be implicated in PDB renoprotection.

Next, we investigated the molecular mechanisms through which GAS5 and miR-21 regulated downstream effectors in renal cells. We focused on PPARα, a member of the nuclear receptor superfamily that has been identified as a major regulator of lipid metabolism [9]. A recent study based on the network pharmacology-based analysis conclusively showed that PPARα was a PDB target protein [4]. Furthermore, previous reports revealed that miR-21 can negatively regulate PPARα expression in many organs [12, 14, 24]. In line with these findings, our results indicated that PPARα mRNA and protein expressions were significantly lower in HG–induced MPC5 cells and STZ-induced renal tissue as compared to normal cells and tissues. On the contrary, 12 weeks of PDB and flavone treatment diminished the effects. As expected, pretreatment of MPC5 cells with si-GAS5 or miR-21 mimic significantly inhibited PPARα mRNA and protein expression in the presence of PDB and flavone under HG conditions, hence blocking the effect of PDB and flavone on PPARα expression. Accordingly, we inferred that GAS5 and miR-21 may be involved in the regulatory effect of PDB and flavone on PPARα. Another member of the PPARs family, PPARγ, has been reported to repress renal fibrosis by alleviating extracellular matrix (ECM) accumulation and the course of DN [38, 39]. Our data indicated that PPARγ mRNA and (or) protein levels in HG–induced MPC5 cells did not change significantly in response to miR-21 overexpression, GAS5 knockdown, or miR-21 inhibition, suggesting that PPARα rather than PPARγ is implicated in the renoprotective effect.

Generally, the aforementioned data inspired us to explore the specific interactions among GAS5, miR-21, and PPARα during the administration of PDB and flavone to DN mice. There is growing evidence that lncRNAs interact with miRNAs and repress reciprocal expression. LncRNAs contain the base complementary region of miRNAs and can act as a competing endogenous RNA (ceRNA) to miRNAs or function as a potent endogenous “sponge” of miRNA. On the contrary, miRNAs can inhibit lncRNAs via argonaute 2 (Ago2)-mediated pathway and may trigger the degradation of lncRNAs [19, 40–42]. Thus, lncRNAs may sequester miRNAs, decreasing their binding to target genes and modulating the derepression of their target genes. Zhang and colleagues discovered a regulatory loop of reciprocal repression between GAS5 and miR-21 expression in breast tumor specimens and cell lines [19]. However, our results indicated that miRNA-21 overexpression could suppress GAS5 expression. Consequently, GAS5 knockdown did not directly affect miR-21 levels but induced a reduction in PPARα gene and protein levels, which is inconsistent with the aforementioned findings, probably due to discrepancies in disease nature, pathogenic conditions, and pathologic processes between studies.

In this study, we identified potential PPARα and GAS5 binding sites in the miR-21 promoter region using bioinformatics prediction tools. Based on the importance of GAS5 and miR-21 in renal tissue and cells, as well as their relationship with PPARα, a dual-luciferase reporter gene assay ascertained that PPARα was a direct target of miR-21 and that lncRNA GAS5 functioned as a ceRNA to promote PPARα expression in renal cells by competing with PPARα for miR-21 binding, which may offer a novel regulatory mechanism among GAS5, miR-21, and PPARα, a finding similar to recent results [43, 44]. This implies that GAS5 is an important modulator of miR-21-targeted PPARα gene and protein expression. Furthermore, a recent study uncovered evidence that the lncRNA MALAT1 regulates KRAS expression, a target gene of miR-217, by altering the spatial distribution of miR-217, particularly by changing the nucleus/cytoplasm ratio of miR-217 [44]. Therefore, the in-depth mechanism for the lncRNA–miRNA–mRNA transregulatory network in our study merits further investigation.

Substantial evidence from previous reports indicates that oxidative stress, fibrosis, and inflammation are involved in the pathogenesis of DN [45, 46] and that they are all interrelated [47–49]. According to research, miR-21 can contribute to renal fibrosis and inflammation by silencing metabolic pathways, whereas its inhibition or deletion reduces renal inflammation under diabetes conditions, increasing the efficacy of treating diabetic kidney injury [12, 37]. Therefore, we focused on the effects of PDB and flavone on oxidative stress, fibrosis, and inflammation of DN and the key role of GAS5 and miR-21/PPARα signaling during this process.

Both experimental and clinical studies have demonstrated that diabetes is associated with elevated oxidative stress levels, primarily attributed to mitochondrial overproduction of ROS. This oxidative imbalance plays a pivotal role in the pathogenesis and progression of DN [50]. In the present study, the biochemical oxidative stress profile as manifested in HG–induced MPC-5 cells showed an obvious increase in intracellular ROS levels when compared to normal MPC-5 cells. Notably, PDB and flavonoid treatment, as observed in HG + P and HG + F groups, abrogated oxidative stress, which was reflected by the significantly low ROS levels when compared to the HG group. Further investigations are required to determine whether the antioxidant property of PDB is attributed to the scavenging of hydroxyl radicals and superoxide anions.

TGF-β is considered a central profibrotic mediator in ECM synthesis and renal fibrosis. Unlike the other two TGF-β isoforms, TGF-β1 is produced in all types of renal cells [51]. Emerging evidence shows that ligand-induced activation of TGF-β1-type II receptor complexes activates Type I receptor serine/threonine kinase and extensively stimulates the downstream SMAD complexes. The heteromeric complexes then translocate into the nucleus and regulate the transcription of target genes including Collagen I, Collagen III, and fibronectin [52]. Accordingly, TGF-β1/SMAD are considered therapeutic targets for renal fibrosis. Reports indicate that TGF-β1 upregulates the miR-21 level via a SMAD3-dependent mechanism during the course of renal fibrosis [53]. However, overexpression of miR-21 in kidney cells promotes TGF-β1 protein level and magnifies the HG–induced abundance of fibrotic markers [54], which indicates that miR-21 may act in a feed-forward loop, thereby enlarging TGF-β1 signaling during renal imaging. According to the current findings, STZ-induced DN mice and HG–induced MPC5 cells resulted in a remarkable enhancement of TGF-β1 levels in serum or urine and supernate as indicated by ELISA, as well as activation of downstream SMAD3 in the DN group compared to the control group. Interestingly, PDB and flavonoid treatment diminished TGF-β1 and SMAD3 levels.

Emerging evidence proved that chronic inflammation had a role in the pathogenesis of DN [55, 56]. Consequently, ELISA was used to measure the levels of inflammatory-related mediators such as IL-1β, IFN-γ, TNF-a, and IL-6 in the serum and urine of DN mice or HG–induced MPC5 cells. The results revealed that the levels of the biochemical parameters were higher in the DN and HG groups, which was consistent with previous studies [49, 56]. Nevertheless, PDB and flavonoid treatment resolved these aberrations by attenuating the altered levels of IL-1β, IFN-γ, TNF-a, and IL-6, implying anti-inflammatory efficacy. Taken together, these results demonstrated that PDB and flavonoids have therapeutic antioxidative, antifibrotic, and anti-inflammatory effects on the onset and progression of DN *in vitro* and *in vivo*.

However, additional investigations of the molecular mechanism revealed that overexpression of miRNA-21 using its mimic or knockdown of GAS5 via its si-GAS5 in podocytes abrogated the inhibitory effect of PDB and flavonoid on the inflammatory mediators, oxidative stress, and fibrotic parameters. We identified that GAS5 acted as a miR-21 ceRNA, competed with PPARα for miR-21 binding, preventing the suppression of its target gene PPARα. Furthermore, recent studies have revealed that activating PPARα by knockout or repression of miR-21 had antioxidative, antifibrotic, and anti-inflammatory effects in DN or other microvascular complications of diabetes, suggesting that PPARα activation could be an effective therapeutic target for diabetic renal or retinal injury [12, 57]. Collectively, these results indicate that the inhibitory effect of PDB and flavonoid on renal inflammation, oxidative stress, and fibrosis was mediated via GAS5 and miR-21/PPARα signaling, which will need to be verified in future studies.

## 5. Conclusions

**Figure 9:**
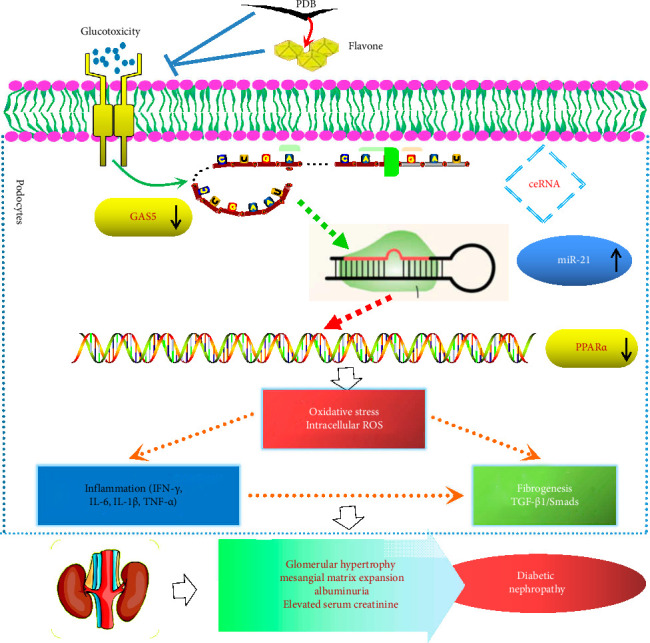
A schematic representation of the proposed mechanisms for *Potentilla discolor* Bunge ameliorating streptozotocin-induced diabetic nephropathy in mice via GAS5 and miR-21 regulation.

In conclusion, the present study shows that PDB has a considerable nephroprotective effect and good potency for treating DN, at least in part, by suppressing renal oxidative stress, fibrosis, and inflammation via GAS5 and miR-21/PPARα signaling (Figure 9). Furthermore, flavone is supported as an active component in PDB. This study also contributes to a better understanding of the molecular mechanisms in DN mice, which may provide novel evidence that PDB has a potential therapeutic benefit in preventing diabetes-induced kidney injury. However, its use in human subjects requires additional clinical evaluation.

## Figures and Tables

**Table 1:** Sequence of siRNAs and miRNA mimics and inhibitors.

Oligonucleotides	Sequence (5′ ⟶ 3′)
si-NC	UUCUCCGAACGUGUCACGUdTdT
si-GAS5	CCUCUGUGAUGGGACAUCUTT
Mimic-NC	UUGUACUACACAAAAGUACUG
Inhibitor-NC	CAGUACUUUUGUGUAGUACAA
miR-21 mimic	UAGCUUAUCAGACUGAUGUUGA
miR-21 inhibitor	UCAACAUCAGUCUGAUAAGCUA

**Table 2:** The primer sequence for the dual-luciferase reporter gene assay.

Primer	Sequence
wt-GAS5	Forward: GACCGCGATCGCGAGTCCAACTTGCCTGGACC
	Reverse: CTTAGTTTAAACGACCCTTTCAAGCAGTAAGC

mut-GAS5	Forward: ATGGAGAGTCGGCTTCTGATGACTGTGTGGA
	Reverse: CATCAGAAGCCGACTCTCCATACCTTTAAAA

wt-PPARα 3′-UTR	Forward: GACCGCGATCGCCACAGCACTCTACGTTGCGT
	Reverse: CTTAGTTTAAACCTGCCATGCCCACACGCTG

mut-PPARα 3′-UTR	Forward: TT CGAGAATAGTTTGTATTCGAATCCCATCAC
	Reverse: TCGAATACAA ACTATTCTCGAATGTTCTCAG

**Table 3:** The primers used in the study.

Primer	Sequence
PPARα	Forward: GAACTGACGTTTGTGGCTGG
	Reverse: CTGGAGAGAGGGTGTCTGTG

PPARγ	Forward: GAAGCGGTGAACCACTGAT
	Reverse: TTCCATCACGGAGAGGTCCA

GAS5	Forward: TGTGGACCTCTGTGATGGGA
	Reverse: ACATTGCGCTCGCTCTGTTA

miR-21	Forward: TAGCTTATCAGACTGATGTTGA
	Reverse: TCAACATCAGTCTGATAAGCTA

β-actin	Forward: CAGCGGAACCGCTCATTGATGG
	Reverse: TCACCCACACTGTGCCCAACGA

U6	Forward: CTCGCTTCGGCAGCACA
	Reverse: AACGCTTCACGAATTTGCGT

## Data Availability

The data used to support the fundings of this study are available from the corresponding author upon request.

## Nomenclature

DN: Diabetic nephropathy
PDB: *Potentilla discolor* Bunge
GAS5: LncRNA growth arrest-specific 5
miR-21: microRNA-21
FBG: Fasting blood glucose
HG: High glucose
DM: Diabetes mellitus
ESRD: End-stage renal disease
CHM: Chinese herbal medicine
PPARα: Peroxisome proliferator-activated receptor alpha
lncRNAs: Long noncoding RNAs
IL-1β: Interleukin-1β
INF-γ: Interferon-γ
TNF-a: Tumor necrosis factor-a
ECM: Extracellular matrix
ceRNA: Competing endogenous RNA
